# Are there foetal extracellular vesicles in maternal blood? Prospects for diagnostic biomarker discovery

**DOI:** 10.1007/s00109-022-02278-0

**Published:** 2022-12-20

**Authors:** Petra Adamova, Robyn R. Lotto, Andrew K. Powell, Iain M. Dykes

**Affiliations:** 1 School of Pharmacy and Biomolecular Sciences, Liverpool John Moores University, Byrom St, Liverpool, L3 3AF UK; 2 Liverpool Centre for Cardiovascular Science, Liverpool John Moores University, Liverpool, UK; 3 School of Nursing and Allied Health, Liverpool John Moores University, Tithebarn St, Liverpool, L2 2ER UK

**Keywords:** Extracellular vesicle, Exosome, Microvesicle, Trophoblast, Placenta, Congenital disease, Diagnostics

## Abstract

Prenatal diagnosis of congenital disease improves clinical outcomes; however, as many as 50% of congenital heart disease cases are missed by current ultrasound screening methods. This indicates a need for improved screening technology. Extracellular vesicles (EVs) have attracted enormous interest in recent years for their potential in diagnostics. EVs mediate endocrine signalling in health and disease and are known to regulate aspects of embryonic development. Here, we critically evaluate recent evidence suggesting that EVs released from the foetus are able to cross the placenta and enter the maternal circulation. Furthermore, EVs from the mother appear to be transported in the reverse direction, whilst the placenta itself acts as a source of EVs. Experimental work utilising rodent models employing either transgenically encoded reporters or application of fluorescent tracking dyes provide convincing evidence of foetal-maternal crosstalk. This is supported by clinical data demonstrating expression of placental-origin EVs in maternal blood, as well as limited evidence for the presence of foetal-origin EVs. Together, this work raises the possibility that foetal EVs present in maternal blood could be used for the diagnosis of congenital disease. We discuss the challenges faced by researchers in translating these basic science findings into a clinical non-invasive prenatal test.

## Introduction

Approximately 3–5% of pregnancies are complicated by congenital anomalies [1]. Early diagnosis improves clinical outcomes [2], yet current screening tools are imperfect. For example, up to 50% of congenital heart defects remain undiagnosed until after birth [3]. Non-invasive methods of screening, which involve a maternal blood test, are increasingly being adopted into clinical practice [4], leading to a marked decrease in the utilisation of invasive diagnostic procedures such as amniocentesis and chorionic villus sampling [5].

Extracellular vesicles (EVs) are small membrane-bound particles produced by many cell types which function in paracrine and endocrine signalling [6–8]. EVs can be detected in most biological fluids, including blood, amniotic fluid and mammary gland secretions [6, 9]. It is reasonably well established that the pre-implantation embryo communicates with the endometrium via EVs [10], whilst the early post-implantation embryo produces EVs that serve to dampen the maternal immune response and to remodel the maternal vascular system [11]. The placenta itself is a source of EVs and a number of recent reviews have discussed the role of these placental-derived EVs in mediating functions such as regulating maternal physiology, immune function and influencing the timing of birth [12–18]. Placental-derived EVs within maternal blood have been proposed as biomarkers for placenta-related diseases such as pre-eclampsia and gestational diabetes mellitus [17, 19, 20].

Experimental evidence from animal models indicates that, in addition to producing EVs, the placenta can transport EVs derived from the mother or foetus between the two circulations. These findings are supported by clinical evidence. Here, we critically assess the evidence for communication across the placenta and the potential of foetal-derived EVs as biomarkers for congenital disease.

## Development and structure of the placenta

The placenta is a temporary organ of pregnancy, forming the interface between mother and foetus and functioning primarily to provide the latter with sustenance required for growth whilst also protecting the foetus from harmful agents [21, 22]. The placenta begins to form shortly after the blastocyst implants within the maternal endometrium, developing from the extra-embryonic part of the blastocyst known as the trophoblast [23–26]. However, it is not until the end of the first trimester in human pregnancies that the mature form is attained and blood supply to the placenta is established [27].

**Fig. 1:**
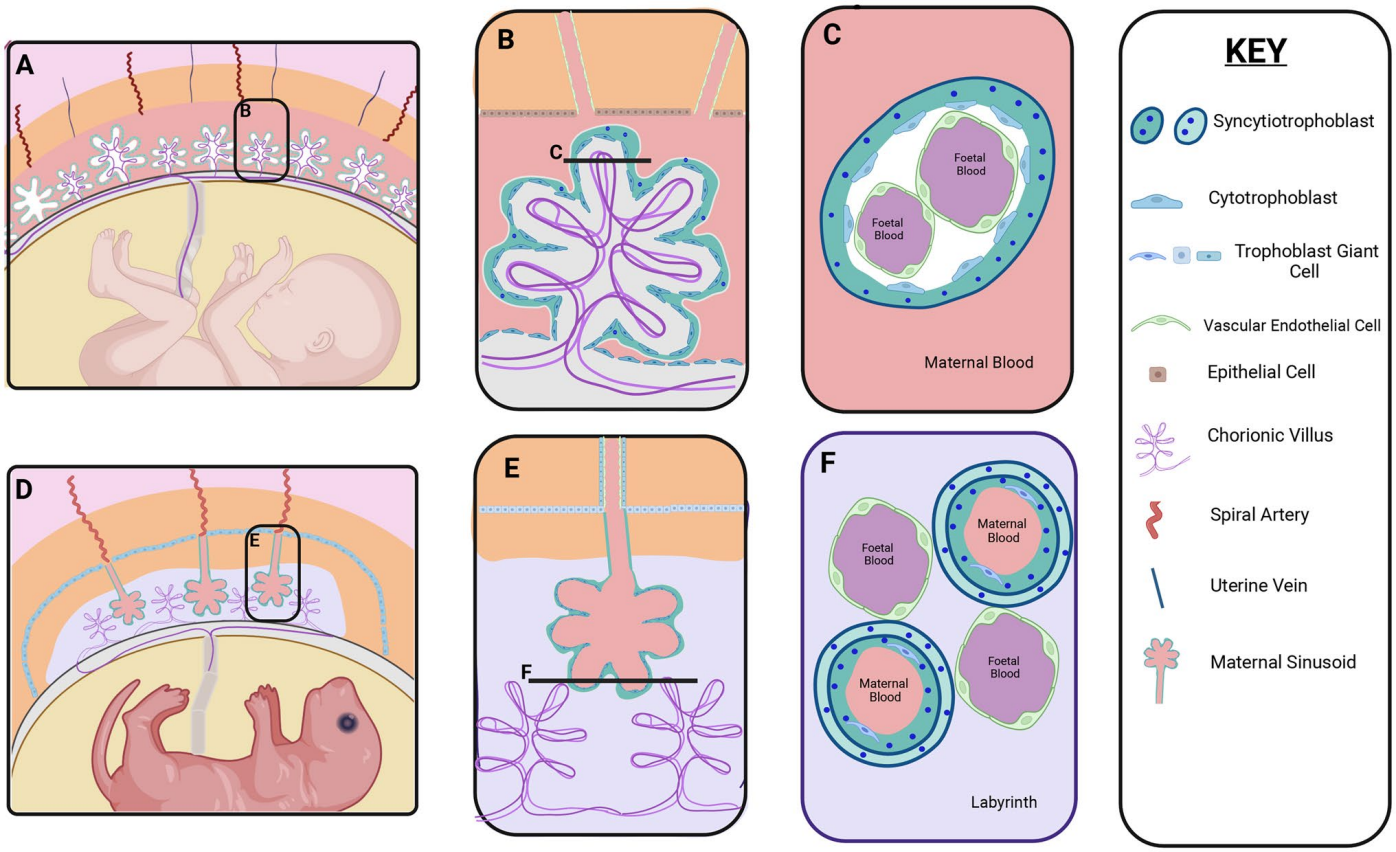
**Anatomy of the placenta**. In both humans (**A**–**C**) and rodents (**D**–**F**), the syncytiotrophoblast forms a barrier separating maternal blood (pink) from foetal blood (purple). In humans, the syncytiotrophoblast surrounds the foetal blood vessels which are located in villi projecting into the maternal blood space. In the rodent placenta, the syncytiotrophoblast surrounds the maternal blood space and separates this from foetal capillaries within a structure known as the labyrinth

In humans [21, 26, 28, 29], foetal blood from the umbilical arteries enters capillaries located within 30–40 chorionic villi which project into an intervillous space bathed in maternal blood (Fig. 1A). This maternal blood is supplied by spiral arteries which empty directly into the intervillous space (Fig. 1A, B). This process is dependent on proteases secreted by the trophoblast, which degrade the endothelial walls of maternal arterioles so that blood can pass unimpeded into the intervillous space [30].

The placental barrier is formed by the syncytiotrophoblast, a multinucleated layer that forms the walls of the chorionic villi, surrounding the foetal vascular endothelium (Fig. 1B, C). As the syncytium has no cell junctions, it forms a barrier separating the two circulatory systems. The syncytiotrophoblast facilitates limited exchange: small molecules (< 600 Da) can diffuse across whilst the transfer of larger molecules can occur only if mediated by specific transport mechanisms [31]. The syncytium is being constantly worn away and it is replenished by the cytotrophoblast, an internal layer of proliferating mononucleated progenitor cells (Fig. 1C) [24]. Foetal blood is separated from maternal blood both by the endothelial layer of the foetal capillary and by the two layers of the trophoblast (Fig. 1C). All of these are foetal cells: there is no maternal cell barrier separating the two circulations. Particles transferred from foetus to mother must pass first through the endothelium, and then through the cytotrophoblast, and finally through the syncytiotrophoblast. Particles transferred from mother to foetus must pass in the opposite direction. It is important to note that the initially continuous cytotrophoblast layer becomes discontinuous as pregnancy progresses. This has a direct consequence on particle transfer, as a full-term placenta has just two continuous layers (the syncytiotrophoblast and the foetal endothelial cells) [32].

There is considerable diversity in placental anatomy within mammals and as a result there is no perfect animal model of placentation [26]. Nevertheless, much of the evidence for transport across the placenta comes from studies of rodent models; therefore, it is important to note that there are anatomical differences between rodent and human placentae. In rodents [28, 29, 33, 34], the spiral arteries do not empty directly into a cavity, but instead are connected to maternal sinusoids, syncytial-lined channels which carry the blood to the site of exchange (Fig. 1D, E). The foetal blood vessels form structures similar to the human villi, but these are not lined with syncytium (Fig. 1E, F). Exchange takes place within a structure called the labyrinth, in which a network of foetal and maternal vessels comes into close apposition (Fig. 1E, F). Thus, in common with the human placenta, the syncytiotrophoblast forms the main barrier of exchange, but in rodents this layer surrounds the maternal rather than the foetal blood (Fig. 1F). In rodents, the syncytium is a double layer, and the mononuclear stem cells, known as trophoblast giant cells (TGCs) [35], do not form a complete barrier (Fig. 1F). In rodents, there are 3 subtypes of TGCs, each with a unique localisation: spiral artery TGCs, maternal blood canal TGCs and TGCs within the sinusoidal spaces of the labyrinth layer [35].

## Extracellular vesicles

### Extracellular vesicles produced by the placenta

EV is a collective term for a heterogeneous population of vesicles of different origins and morphology. Three subclasses of EVs, distinguished by size and origin, are generally recognised in the literature: exosomes (nanovesicles), microvesicles (ectosomes) and apoptotic bodies [9, 36].

**Fig. 2:**
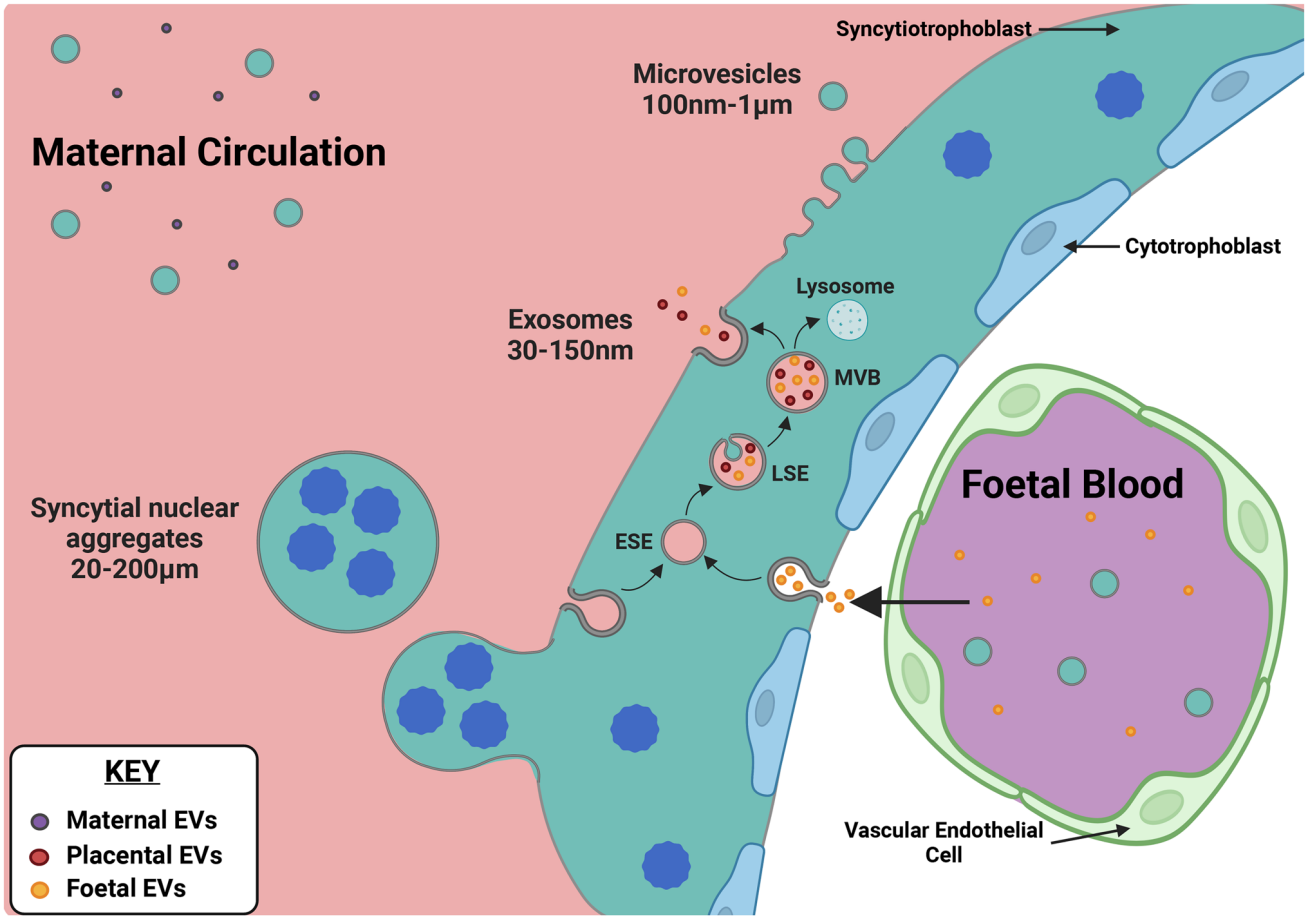
**Trafficking and production of extracellular vesicles by the placental syncytiotrophoblast**. Three classes of EVs produced by the syncytiotrophoblast are detectable in maternal blood. Exosomes (30–150 nm) originate within the endosomal pathway, whilst microvesicles (100–1000 nm) are produced by blebbing from the plasma membrane. Syncytial nuclear aggregates are a class of large vesicles unique to the trophoblast which contain nuclei. The placenta releases endogenous EVs, but also traffics EVs from the foetal to the maternal circulation. It is hypothesised that foetal EVs are endocytosed and processed through the endosomal pathway. ESE, early sorting endosome; LSE, late sorting endosome; MVB, multivesicular body

The smallest EVs are known as exosomes (size ~30–150 nm) [7, 8, 37]. Exosomes are derived from the fusion of multivesicular bodies (MVBs) with the plasma membrane, releasing their intraluminal vesicles (ILVs) as exosomes (Fig. 2). The process of exosome biogenesis begins by endocytosis and plasma membrane invagination leading to the formation of an early sorting endosome (ESE) [38] and then a late sorting endosome (LSE). Secondary invagination of the LSE membrane forms ILVs, thus creating the MVB [39, 40]. MVBs can either be targeted for lysosomal degradation through fusion with an autophagosome or they can be transported to the plasma membrane for exosome release. At the plasma membrane, the cytoskeletal and microtubular network on the luminal side of the plasma membrane and the MVB-docking proteins fuse the MVB with the membrane leading to exocytotic release of ILVs into the extracellular fluid as exosomes [40].

Intermediate-sized vesicles are known as microvesicles (MVs) or ectosomes (size ~100nm–1 μm) [37, 41]. MV formation is poorly understood; however, they are believed to originate by the direct outward budding of the plasma membrane (Fig. 2) [41]. Importantly, MVs overlap in size with exosomes and cannot be separated from them by size-based purification methods such as ultracentrifugation and size exclusion chromatography [42, 43].

The largest class of EVs are apoptotic bodies. Apoptotic bodies (size ~50nm–5 μm), as the name suggests, are a product of apoptosis [9, 37, 44, 45]. Although they might at first appear to represent the debris of dying cells, signalling by apoptotic bodies has been demonstrated in recent studies [46]. The composition of these EVs is very different to exosomes and MVs as they contain intact organelles and chromatin [37].

The trophoblast, which is derived from embryonic tissue (“Development and structure of the placenta”), has been shown to produce EVs, and these are secreted into the maternal circulation (Fig. 2) [47]. These EVs include exosomes and MVs but not apoptotic bodies. The syncytiotrophoblast, like other epithelial tissues, is constantly undergoing turnover with aged tissue being shed and replaced by new tissue from the cytotrophoblast daughter cells. However, as the trophoblast is a multinuclear syncytium, it cannot fragment into small particles to produce apoptotic bodies as a single cell would [24]. Instead, clusters of aged nuclei are extruded and released as large multinuclear structures known as syncytial nuclear aggregates (SNAs size 20–200+μm) (Fig. 2) [24, 48–51]. Whether SNAs are the placental equivalent of an apoptotic body remains controversial [52]. These large particles can be detected in maternal blood, carry mRNA [49] and express some of the biomarkers found in smaller EVs.

EV subtype classification and isolation is often very difficult because particles overlap in size and no specific markers of EV subtypes are currently available [53]. Some authors have proposed a further subdivision of these subtypes (e.g. classical vs. non-classical exosomes) [8]. Indeed, it is sometimes unclear precisely which subclass of EV some older papers are describing. The term syncytiotrophoblast-derived microvesicle (STBM) is sometimes used as a general term for undefined placental EVs. For this reason, the International Society for Extracellular Vesicles (ISEV) recommends that the general term EV should be used and that specific details of isolation procedures and EV characterisation be reported [53]. In this review, we will primarily be discussing exosomes and MVs and, unless otherwise specified, we will use the general term EV to describe these collectively.

### Extracellular vesicle transport across the placenta

It is likely that the foetal blood contains EVs originating from the developing organs, which may provide information on congenital disease. The mechanisms by which EVs cross the placenta are yet to be fully understood. The multinucleated syncytiotrophoblast presents an impermeable barrier lacking intercellular junctions or pores. It therefore seems most likely that EVs must pass through the syncytium itself, being taken up on one side and subsequently released on the other. To our knowledge, there have been no studies investigating how EVs are taken up by the placenta itself. However, a number of reports describe the mechanism of uptake of EVs into target cells (reviewed in [54, 55]). For example, placenta-derived EVs enter target cells in the uterus by endocytosis and are trafficked to early and late endosomes [56]. Thus, it seems possible that this pathway could allow for trafficking of EVs across the syncytiotrophoblast and release on the opposite surface from MVBs (Fig. 2). It is possible that vesicle surface proteins or glycoproteins/proteoglycans interact with trophoblast membrane receptors prior to endocytosis and this may limit which EVs are taken up. There is very little data on whether or how such transported EVs may be processed prior to release. For example, placental EVs are known to express tissue-specific surface markers (discussed below), but whether trafficked EVs originating from the foetus proper take up these markers is not known.

### Extracellular vesicle biomarkers

EVs carry a cargo consisting of RNA and soluble proteins and in addition are enriched in specific transmembrane and membrane-associated proteins and extracellular glycans at the vesicle surface. Surface proteins tend to be involved in aspects of vesicle biogenesis whilst cargo molecules are actively loaded into vesicles from a number of sources. However, many proteins are shared by the different EV subtypes and unique biomarkers are hard to identify.

The endosomal sorting complex required for transport (ESCRT) pathway regulates membrane budding and separation in a number of cellular processes including both the formation of ILVs during exosome biogenesis and MV budding from the plasma membrane [57, 58]. ESCRT complexes also have a secondary function in cargo loading of proteins tagged with ubiquitin [57]. Common ESCRT biomarkers of EVs include ALG-2 interacting protein-X (ALIX) and tumour susceptibility gene 101 (TSG101).

The tetraspanins are four-pass transmembrane proteins that mediate protein–protein interactions. Tetraspanins form clusters within the membrane associated with various proteins and these clusters later bud off the membrane to form vesicles [57, 58]. The tetraspanin termed cluster of differentiation 63 (CD63) is enriched in the endosomal pathway, specifically within ILVs of MVBs, and therefore is generally regarded as a specific marker of exosomes [8, 59]. However, tetraspanins are also present in the plasma membrane and may also be detected in MVs [58, 60]. Other tetraspanins enriched in EVs include CD81 and CD9.

During exosome biogenesis, ESEs communicate with the mitochondria, the endoplasmic reticulum (ER) and the trans-Golgi network, obtaining cargo from these sources [7, 58]. EVs also carry a cargo of RNA, particularly microRNA (miRNA), which are likely to function in the regulation of gene expression in target tissues [61, 62]. This function remains controversial [63], but nevertheless, blood miRNA is potentially useful as a biomarker and many studies have reported potential diagnostic uses [64–66]. Specific mechanisms for loading miRNA into vesicles exist [67]. Thus, EVs can be thought of as vectors to transport signals between foetal and maternal tissues in order to exert a functional effect [68].

## Evidence for EV-mediated communication between the mother and the foetus across the placenta

Pregnancy requires substantial crosstalk and signalling between the mother and the foetus across the placenta, and this is linked to various physiological events during pregnancy. The trophoblast produces hormones that influence the mother, whilst antibodies are transferred from mother to foetus. Evidence is emerging that this bidirectional communication may be mediated in part by EVs. The trophoblast appears to be a source of EVs, and in addition, may permit the transfer of EVs between maternal and foetal circulation. Here, we will review the evidence for EV-mediated communication across the placenta beginning with a discussion of the evidence derived from rodent models, followed by a discussion of evidence from clinical studies.

### Rodent models

#### Use of transgenic reporters

**Fig. 3:**
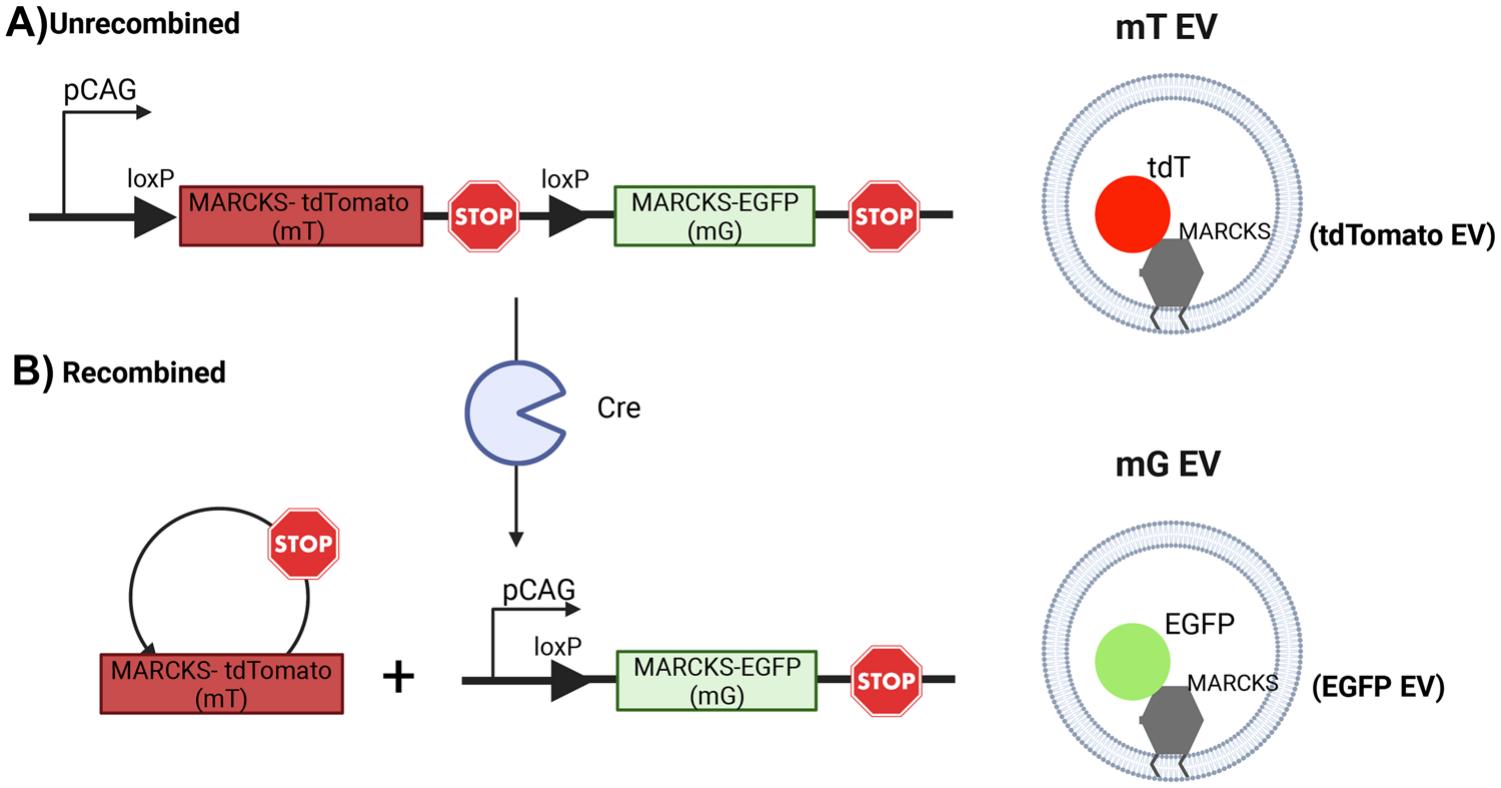
**The mT/mG mouse model expresses fluorescently labelled EVs**. **A** mT/mG mice have been engineered to carry a single copy of a transgene inserted randomly into the genome. The transgene (left) consists of two expression cassettes for the lipid-anchored MARCKS protein fused to a fluorescent reporter (mT: tandem dimer tomato; mG: enhanced green fluorescent protein [EGFP]). The ubiquitously expressed pCAG promoter drives expression. LoxP sites flank the mT cassette. In the unrecombined allele, an upstream STOP codon prevents translation of mG, and thus, EVs express mT (right). **B** The enzyme CRE-recombinase recognises the LoxP sites and mediates recombination of the allele, resulting in excision of the mT cassette. Following recombination, the pCAG promoter drives expression of mG, and thus, EVs express mG (right)

Rodent models have been used in several elegant experiments to examine transport of EVs across the placenta. One strategy has been the use of a transgenic mouse line in which a fluorescent reporter is expressed only in the paternal lineage and is therefore normally lacking in the mother. Detection of this marker in EVs within maternal blood or tissues provides evidence that foetal- or placental-derived EVs have either been released by the placenta or trafficked across it. The mouse line most commonly used in these experiments (the mT/mG mouse; Fig. 3) expresses a fluorescent reporter—either green fluorescent protein (GFP) or tandem dimer tomato (mT)—fused to the N-terminal domain of the myristoylated alanine-rich C-kinase substrate (MARCKS) protein, a widely expressed lipid-anchored membrane protein enriched in EVs [69, 70]. Sheller-Miller et al. [71] mated a wild-type female to a reporter male carrying the mT/mG transgene and immunoprecipitated mT+ EVs from maternal plasma at gestation age E16 using an antibody against tdT. They found that 35% of total maternal plasma EVs were mT + indicating that these EVs were released by a cell of embryonic origin (either foetus proper or placenta). In addition, mT was detected in the cervix and uterus and co-localised with the EV marker CD81, suggesting that these EVs are specifically targeted to these maternal tissues.

The mT/mG reporter mouse carries a floxed allele in which LoxP sites flank the mT coding sequence (Fig. 3) [69]. When cells carrying this allele are exposed to CRE (phage P1 cyclic recombinase), the allele is recombined; thus, the mT sequence is excised from the locus and the resulting cells (and EVs derived from them) express the mG reporter (membrane targeted GFP) instead of mT (Fig. 3). In a clever experiment, Sheller-Miller et al. [71] loaded EVs isolated from the culture medium of the human embryonic kidney line HEK293T in vitro with CRE protein and then intraperitoneally injected these into pregnant wild-type mice carrying mT/mG foetuses at gestation age E13. They reported expression of mG in placenta (demonstrating placental uptake) and foetal tissue membranes (demonstrating trafficking into foetal circulation). This provides evidence that EVs from the maternal circulation had crossed the placenta and delivered their cargo of functional CRE enzyme to foetal cells. Furthermore, mG+ EVs could be detected in maternal plasma, indicating the EVs derived from recombined foetal cells had subsequently been released by the placenta in the reverse direction [71]. Together, this work provides convincing evidence of bidirectional EV-mediated communication.

Nguyen et al. [72] used the same mT/mG reporter mouse. They crossed an mT/mG female with a male line expressing an X-linked constitutive CRE transgene. In this model, all maternal tissue should express endogenous mT, but detection of mG indicates the presence of EVs derived specifically from a cell of female embryonic origin. mG expression was detected in maternal lungs (supporting the tracking dye studies discussed below). Interestingly, Nguyen et al. also provide evidence that CRE+ EVs may have been released by the placenta and induce recombination of the locus in the maternal lungs resulting in endogenous mG expression.

In addition to the work on maternal–foetal communication across the placenta, the mT/mG mouse has been useful in demonstrating EV transport across the blood–brain barrier. For example, Mustapic et al. [73] used the brain-specific Nestin-CRE line to recombine the locus and subsequently demonstrated mG+ EVs in peripheral blood.

One caveat of these studies is that although the mT/mG transgene was inserted into the ROSA26 locus, a widely used strategy for such targeting normally without affecting the mouse [74], mT/mG homozygous mice exhibit a failure in mechanoelectrical signal transduction of auditory hair cells leading to deafness [75]. High levels of mT prevent transmembrane channel-like protein 1 (TMC1), a component of the mechano-transducer channel, localising to stereocilia tips of auditory hair cells. These data suggest that the reporter can interfere with the function of endogenous membrane proteins, raising the possibility that endogenous EV proteins could be affected in this mouse model.

Other transgenic models have been developed which may overcome this problem, for example, a transgenic rat expressing GFP-tagged human CD63. Such fluorescent EVs can be isolated from various bodily fluids in this animal including blood and amniotic fluid [76]. The same group has generated rats expressing tissue-specific CD63-GFP using a SRY-box transcription factor 2 (SOX2) promoter to drive neural-specific expression in the foetus [77]. Furthermore, an inducible reporter has been produced by generating transgenic mice expressing CD9-GFP under the control of a stop-floxed ubiquitous chicken beta-actin (CAG) promoter [78]. This mouse can be crossed with tissue-specific or tamoxifen-inducible CRE lines to produce tissue-specific or temporally controlled expression, respectively [78]. These methods are yet to be applied to foetal-maternal EV communication studies but offer promising alternatives to the mT/mG mouse model.

#### Use of tracking dyes

**Fig. 4:**
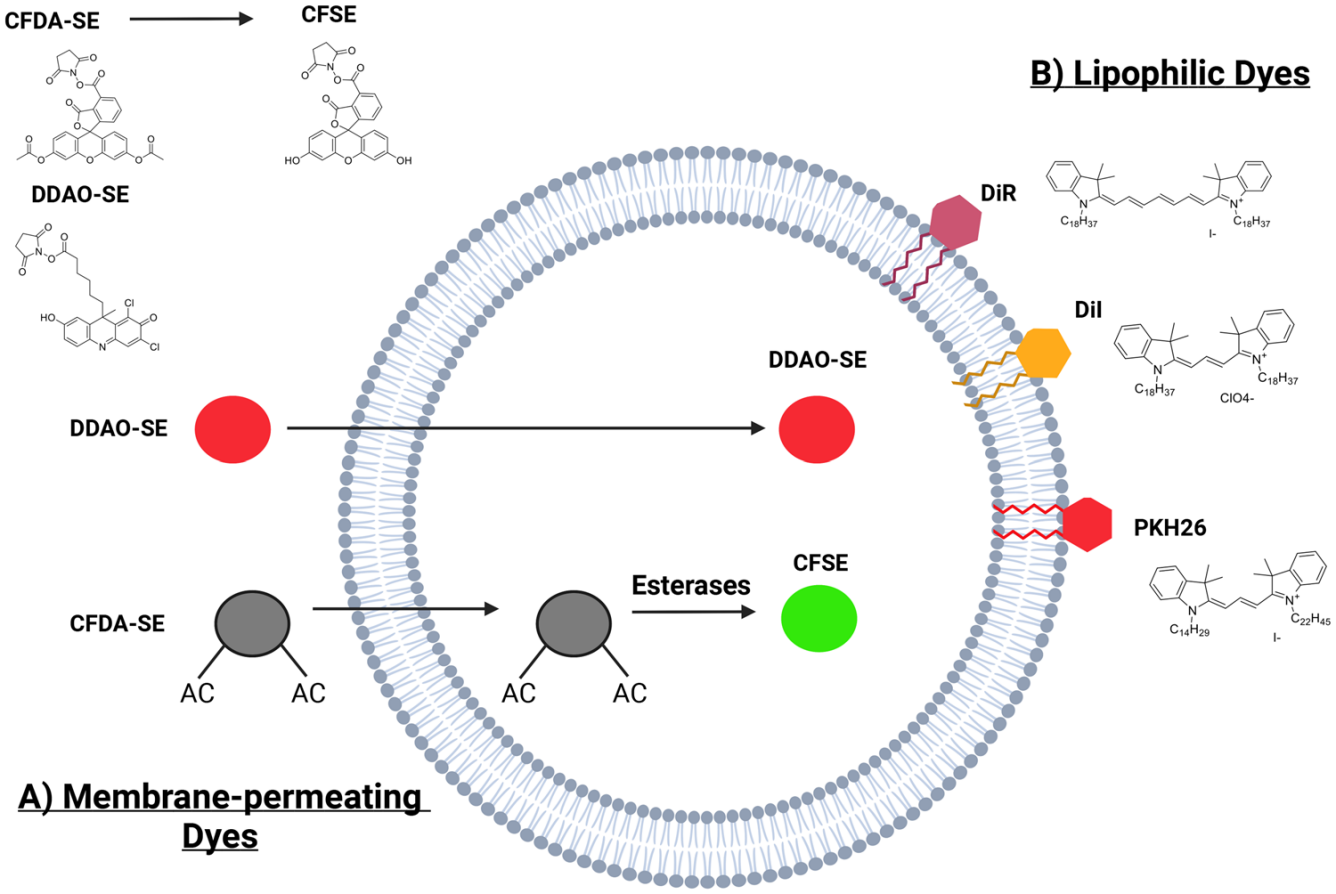
**Tracking dyes commonly used to label EVs**. Two classes of fluorescent dyes are commonly used for EV labelling: membrane-permeating and lipophilic dyes. **A** Membrane-permeating dyes (DDAO-SE and CFDA-SE) are lipophilic molecules that can cross the EV membrane. CFDA-SE is minimally fluorescent in the native state, but is activated once inside the EVs by endogenous esterases which cleave the acetate groups. DDAO-SE, on the other hand, does not require esterases to be activated; instead, it forms covalent attachments to amines both inside and outside of EVs. **B** Lipophilic dyes (DiR, DiI and PKH26) are a family of fluorescent stains for labelling membranes and other hydrophobic structures. The fluorescence of these dyes is enhanced once incorporated into the EV membrane

Because the syncytiotrophoblast is derived from the embryo, the mT/mG transgenic model cannot differentiate between EVs trafficked across the placenta from those originating within it. An alternative strategy, and one that addresses this problem, is to trace the trafficking of labelled EVs injected into the animal. Fluorescent dyes that have been used successfully in these studies include lipophilic dyes (DiI, DiR and PKH26, see Abbreviations for full names) which label all lipid membranes, and membrane-permeable dyes which enter EVs and then react with the amine groups of cargo proteins (DDAO-SE and CFDA-SE) (Fig. 4). CFDA-SE is particularly useful because fluorescence must be induced through cleavage by esterases present within the EV, thus reducing background.

Shi et al. [79] isolated EVs from the maternal blood of pregnant mice and labelled them with DiI before injecting these back into pregnant mice via the tail vein at a number of timepoints (E8.5, E11.5, E14.5). The embryos were then harvested at E16.5, sectioned and imaged. DiI signal was observed in the embryonic heart, placenta and other tissues, suggesting that EVs derived from maternal blood had crossed the maternal-foetal barrier and entered specific foetal tissues. Liu et al. [80] isolated EVs from visceral adipose tissue of pregnant mice, labelled them with DiI and then injected these into the tail vein of pregnant mice at gestation days E8.5 and E11.5. Labelled EVs were detected in the placenta and the embryonic heart on embryonic day E16.5. Sheller-Miller et al. [71] purified EVs from maternal plasma at two gestational ages, E9 and E18. They labelled these EVs with CFDA-SE and then intraperitoneally injected E15 mice three times at 6 h intervals, followed by a final injection 12 h later. They demonstrated fluorescent signals at day E17 in both maternal (cervix and uterus) and foetal tissues (placenta and membranes). Thus, three independent studies have demonstrated trafficking from the maternal blood across the placenta to specific foetal tissues.

Trafficking in the opposite direction across the placenta from foetus to maternal tissue has also been demonstrated. Sheller-Miller et al. [81] extracted EVs from the culture medium of primary human amnion epithelial cells grown in vitro, labelled them with DiR and then injected these into the amniotic fluid of mouse foetuses at E17. They demonstrated fluorescence on the maternal side of the placenta 1 day after injection, as well as targeting to maternal kidneys and uterus. These data provide evidence of both trans-placental communication and of specific targeting to maternal tissues. In addition to the transgenic work described above, Nguyen et al. [72] purified EVs from placental explant medium, labelled with PKH26 and then intravenously injected these into non-pregnant female mice. Thirty minutes after injection, the mice were sacrificed and the lung and liver were harvested. Fluorescent microscopy analysis of the lungs supports the previous finding that placental EVs can traffic to the maternal lung where they appear to be taken up by interstitial macrophages. When EVs derived from the plasma of pregnant and non-pregnant mice were labelled with PKH26 and then injected into the tail vein of non-pregnant recipients, only those EVs from pregnant mice were detected in the maternal lungs [72]. This suggests that foetal-derived EVs present in the plasma of the pregnant mice are able to target maternal tissue. The liver was not targeted in either condition.

Tong et al. [82] explored the specific targeting of foetal EVs to maternal tissue in more detail. They collected EVs from the culture medium of human first trimester placenta (8–12 weeks), labelled these with DDAO-SE and injected them into the tail vein of pregnant mice at E12.5. Ten major organs were imaged at both 30 min and 24 h after injection to monitor targeting. This work demonstrated that EVs are quickly targeted to maternal lungs and liver and later (at 24 h) to the kidneys. Importantly, these authors also report that many maternal organs are not targeted by foetal EVs, including the brain and heart. Furthermore, Tong et al. [82] provide evidence that specific maternal tissues become receptive to foetal EVs during pregnancy: foetal EVs modify the vasoconstrictive behaviour of mesenteric arteries in pregnant but not in control non-pregnant mice.

#### Summary of rodent model data

In summary, work in rodents has demonstrated bidirectional transport across the placenta indicating that foetal EVs may be detectable in maternal circulation. About one-third of EVs in the maternal circulation appear to be derived from either the foetus or the placenta and these EVs demonstrate specific targeting to multiple maternal tissues including the reproductive system (uterus, cervix) and enteric organs (lungs, kidneys, and liver). Some of these tissues appear to become receptive to these EVs only during pregnancy, whilst others are receptive in non-pregnant controls. Other organs, such as the heart and brain, do not appear to be targeted. On the foetal side, maternal EVs are targeted to the heart and placenta. EVs are able to deliver functional protein cargo to both maternal and foetal tissues.

### Clinical evidence

EVs have been isolated from the maternal blood of patients as part of investigations into a number of diseases of pregnancy and these data provide evidence that many of the findings from rodent models are applicable to humans despite structural differences in their placentas (Fig. 1). One caveat of maternal-foetal studies is that together with foetal and maternal EVs, the placenta itself releases placenta-derived EVs which can be detected in the maternal circulation as early as 6 weeks after conception, and their levels continue to increase with gestational age [13, 83].

#### Maternal plasma EV concentration increases during pregnancy

Clinical studies measuring the total EV concentration within the blood have demonstrated that pregnant women have a higher concentration of EVs than non-pregnant women, and that EV concentration increases during the course of a pregnancy. Salomon et al. [84] measured the EV protein concentration isolated from plasma in first trimester pregnant women and found it to be more than 50-fold greater than non-pregnant controls with this increasing to a 100-fold difference by the third trimester. When looking at EV number, they found this increased ~5-fold between first and second trimesters, and ~13-fold by the third trimester. The same group showed that EV numbers increase progressively during weeks 6–12 of the first trimester [83]. Sabapatha et al. [85] found ~13-fold increase in blood plasma EV protein content between third trimester pregnant women and non-pregnant controls. Finally, these results were further supported by Pillay et al. [86] who showed that total CD63+ EV numbers increase between early (<33 weeks) and late (>34 weeks) third trimester pregnancies.

#### Evidence for placental EVs in maternal circulation

**Fig. 5:**
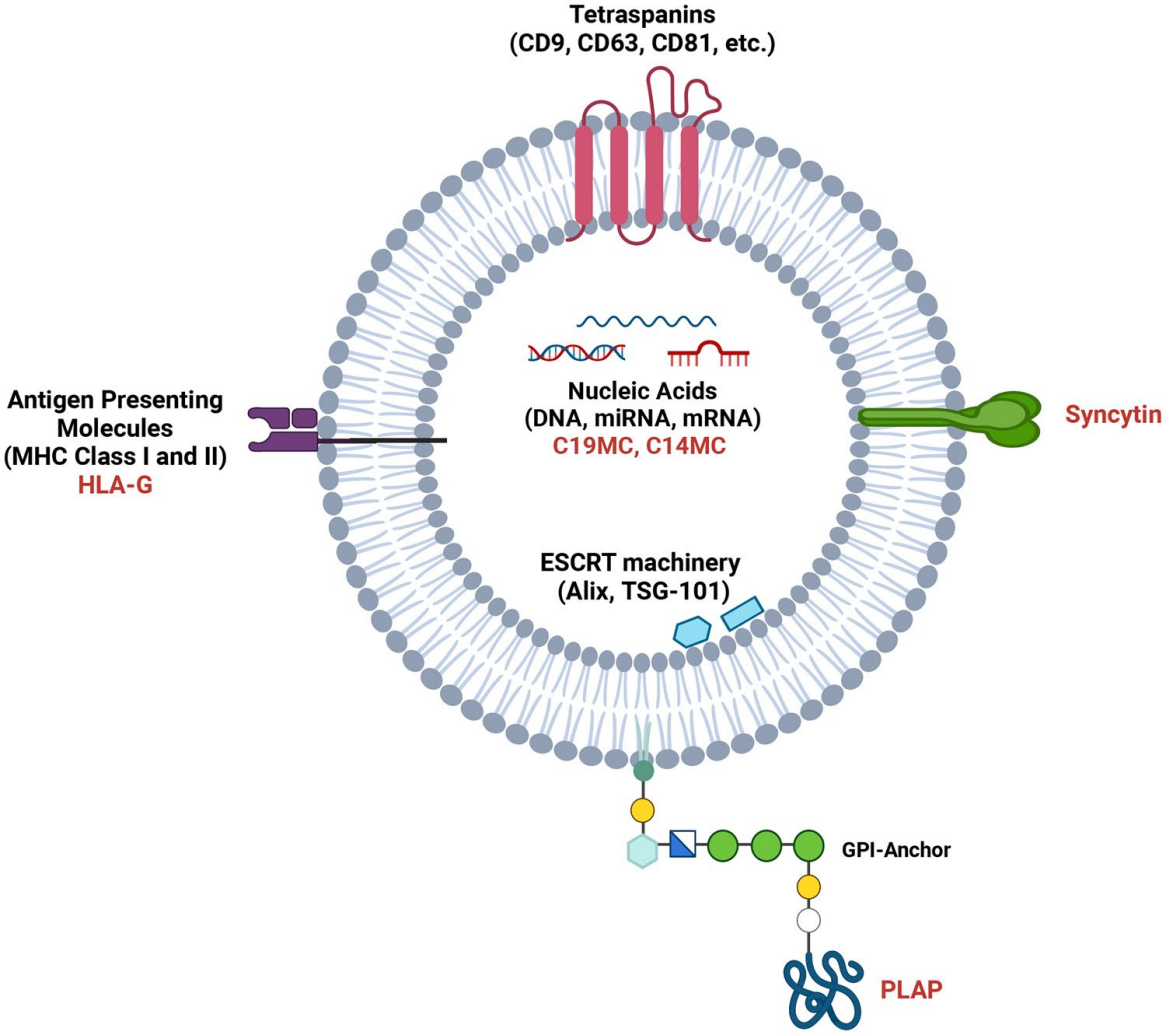
**Endogenous markers of placental EVs**. EVs express various proteins, glycoproteins and lipid-associated molecules at or near the membrane. These include tetraspanins (CD9, CD63, CD81), antigen-presenting molecules (MHC class I and class II) and ESCRT complex proteins. EVs also carry a cargo including nucleic acids (DNA, miRNA, mRNA). Placental-specific EV markers are shown in red and include HLA-G, syncytin, the lipid-anchored phosphatase PLAP and miRNA expressed from the C19MC and C14MC clusters

The studies described above demonstrate that maternal blood EV concentration increases during pregnancy, but do not determine the origin of these EVs. The origin could be maternal tissue (secreted directly into maternal blood) or foetal tissue (secreted into foetal blood and having crossed the placenta into maternal blood) or might be produced by the placenta itself. In order to differentiate between these possibilities, specific endogenous markers for the placenta or other foetal tissues are needed to identify foetal-origin EVs within the maternal blood. Fortuitously, a number of proteins and miRNAs demonstrate a placental-specific expression and can be used to identify EVs released by the placenta. Many of these are found only in humans and other primates and therefore cannot be used in rodent studies. It is important to note that it is currently unclear precisely how foetal EVs are trafficked across the placenta and it is conceivable that these foetal EVs acquire placental biomarkers during this process. Here, we will focus on the best known endogenous placental markers: placental alkaline phosphatase (PLAP), syncytin, human leukocyte antigen-G (HLA-G) and placental-specific miRNA (Fig. 5).

##### Placental alkaline phosphatase (PLAP)

Placental alkaline phosphatase (PLAP, gene name ALPP) is a glycosylphosphatidylinositol (GPI)–anchored membrane protein, which is secreted by the syncytiotrophoblast [87]. PLAP is a member of the human alkaline phosphatase family, which is a multigene family composed of four alkaline phosphatase isoenzymes [88]. A number of studies have demonstrated PLAP+ EVs in maternal blood. PLAP is present both in small EVs of a size consistent with exosomes and microvesicles [83, 84, 86, 89, 90], and in larger SNAs [91–96].

In order to measure the proportion of EVs within the maternal circulation derived from the placenta, most studies have combined a measurement of PLAP protein (such as ELISA [83, 84, 86] or flow cytometry [89]) with a measurement of total EV number (either determined directly by nanoparticle tracking analysis (NTA) [83, 84, 89, 90] or determined by measuring the amount of the general EV marker CD63 [86]). The data show that whilst the levels of PLAP+ EVs increase throughout pregnancy, this reflects a more general increase in EVs in the maternal circulation and the ratio of PLAP+ to PLAP− EVs remains constant during the first and second trimesters before increasing dramatically at the start of the third [84, 86]. Thus, the increase in EVs during pregnancy may result from both foetal and maternal sources.

PLAP expression is restricted to humans and great apes [97, 98]. Mice do not possess a placental isoform [97]. However, a transgenic mouse has been generated expressing human PLAP and this appears to be expressed in all embryonic tissues [97]. There are no studies to date reporting its use for EV tracking, and this would be useful to investigate in the future.

##### Syncytin

The membrane-expressed glycoproteins syncytin-1 and syncytin-2 (gene names *ERVW1* and *ERVW2*) mediate membrane fusion events leading to the formation of the syncytiotrophoblast, hence the name [99, 100]. They may also play a role in membrane fusion events related to EV uptake [101]. The mouse genome has two homologues, syncytin-A and syncytin-B [102]. Loss of function of either syncytin or its receptor lymphocyte antigen 6 family member E (LY6E) causes embryonic lethality in mice [103, 104].

The presence of syncytin-1 in human placental EVs was first demonstrated in vitro using ex vivo placental explant cultures [105]. Syncytin-1 co-localises with PLAP in both small and large placental explant EVs [106]. Syncytin+ EVs are also produced by the placental cell line BeWo in vitro [105, 107]. Syncytin-1+ and syncytin-2+ EVs have been isolated from the blood of pregnant women [101, 107] and shown to co-localise with PLAP [101]. In mice, syncytin + EV levels in the blood peak just before birth (E17–E18) before returning to baseline postpartum [98]. This has not been demonstrated in humans but it is known that levels of synctin-2 + EVs are lower in women with pre-eclampsia [108].

##### Human leukocyte antigen-G (HLA-G)

Mechanisms have evolved to prevent immune rejection of the foetus by the mother during pregnancy. One of these is the expression of inhibitory molecules by the invading extravillous trophoblast, which serves to dampen the maternal immune response to foreign (paternal-derived) antigens (reviewed in [13, 109]). Human leukocyte antigens (HLAs) are antigen-presenting cell-surface glycoproteins recognised by T cell receptors expressed by both T cells and NK cells, which function to distinguish self from nonself [110, 111]. The role of EVs in this process has recently been reviewed by Bai [13]. EVs either stimulate or suppress the response of the immune cells depending on receptors expressed by the EV membrane. There are two major classes of HLA genes. Class II genes are expressed only by antigen-presenting cells and recognised by helper T cells (T_H)_) whilst class I genes are expressed by all cells and recognised by cytotoxic T cells (T_c_) [110]. Syncytiotrophoblasts lack classical class I HLAs but express the non-classical HLA-G which binds to inhibitory receptors expressed by NK cells and thus prevents them from attacking foetal-derived cells [112].

HLA-G is present in EVs isolated from placental explant cultures, but is more abundant in EVs from first trimester explants and nearly undetected in term explant-derived EVs [113]. This is consistent with the role of HLA-G in dampening the maternal immune response early in pregnancy. Both small and large HLA-G+ EVs have been isolated from the blood of pregnant women, and levels of these have similarly been found to decrease as pregnancy progresses [96, 114]. However, it should be noted that some tumour-derived EVs also express HLA-G [115]; therefore, HLA-G may be unsuitable as an endogenous placenta biomarker.

##### miRNA

The placenta expresses unique placental-specific miRNAs derived from two clusters, one on chromosome 19 (C19MC), which is primate-specific, and the other on chromosome 14 (C14MC), found in a wider range of mammals including mice [116–118]. Interestingly, these clusters are imprinted, with C14MC miRNA expressed only from the maternal chromosome and C19MC from the paternal [117]. The precise roles of C19MC and C14MC miRNA remain poorly understood; however, it has been noted that they appear to have an antagonistic effect on placental growth. Mice expressing either the ectopic human C19MC paternal-imprinted cluster [119] or with a knockout of the maternal-imprinted C14MC cluster [120] show increased placental growth [121]. A number of studies have suggested that these miRNAs may be present in EVs released by the placenta and that levels increase in maternal blood during pregnancy, before decreasing following birth [122–126]. They have also been detected in umbilical cord blood, suggesting communication from placenta to foetus [127].

#### Evidence for foetal EVs in maternal circulation

The discovery that placental-specific proteins are expressed on the surface of EVs facilitated the antibody-based or immune-based detection and isolation of placental-origin EVs as a sub-population in maternal circulation. As the placenta is a transient organ of pregnancy, such biomarkers are not expressed in adult tissue. It is more difficult to definitively identify EVs originating from the foetus proper within the maternal circulation due to a lack of specific biomarkers. Whilst biomarkers of particular organs can be identified, most are also expressed in the adult. One way around this problem is to search for differences in pregnancies affected by congenital disease. This provides indirect evidence for the transfer of foetal EVs; however, to draw conclusions from these studies one must assume that there are no changes in the mother’s physiology in such cases.

##### Evidence from ex vivo studies for transport across the placenta

In vitro studies of ex vivo placentas derived from term pregnancies have demonstrated that particles of the size of EVs can be transported bidirectionally across the human placenta (reviewed in [128]). In one study, Grafmueller et al. [129] showed that polystyrene beads in the size range 50–300 nm could be transported in both directions across the placenta. The rate of transfer was higher in the foetal-maternal direction. In another study, Wick et al. [130] demonstrated that 240-nm particles could be transported across whilst 500-nm particles could not. Thus, the potential for foetal to maternal transfer exists.

##### Evidence for EVs from the foetal nervous and cardiovascular systems in maternal circulation

There is some evidence that EVs expressing markers reported to be of foetal brain tissue origin can be detected in maternal blood. Marell et al. [131] demonstrated that umbilical cord blood EVs express the glycosylphosphatidylinositol (GPI)–anchored neuronal membrane protein CNTN2 and the neural growth factor brain-derived neurotrophic factor (BDNF), suggesting that foetal blood contains EVs derived from the developing brain. Goetzl et al. [132, 133] demonstrated that CNTN2+ EVs can be purified from maternal plasma as early as 10–19 weeks of gestation and are ~tenfold more abundant in the blood of pregnant women (first and second trimester) compared to non-pregnant controls. Together, this work suggests foetal-maternal transfer, but it should be noted that both biomarkers are also expressed in the adult, whilst CNTN2 is highly expressed in the Purkinje fibres of the heart [134], and is therefore not neural-specific.

The evidence for transfer of cardiovascular EVs comes from two studies reporting changes in cargo expression within EVs isolated in congenital disease. Both studies isolated EVs from maternal blood before profiling RNA expression. Jin et al. [135] identified miRNAs that were differentially expressed in pregnant women carrying a child with ventricular septal defects compared to the control group, whilst Huang et al. [136] identified differences in long non-coding RNA (lncRNA) expression in pregnant women carrying a child with pulmonary stenosis. In another study, Gu et al. [137] identified differentially expressed lncRNA in maternal blood from a mixed cohort of CHD cases (including both ventricular and atrial septal defects as well as Tetralogy of Fallot) versus healthy controls. Although the authors did not isolate EVs from blood, lncRNA are not believed to exist in blood outside of EVs. These studies together provide indirect evidence for foetal EV transfer if we assume no changes in the mother.

##### Evidence for foetal miRNA within maternal circulation

There are a number of studies suggesting a change in miRNA expression in maternal blood associated with the presence of a specific congenital disease. Most studies do not isolate EVs and are therefore analysing both the miRNA loaded into EVs and that which is freely circulating. Examples include changes in congenital heart disease [65, 138], Down’s syndrome [139] and those subsequently shown to have a low birth weight [66]. These changed miRNAs may or may not originate from the foetus.

## Prospects for diagnosis of congenital disease

The most common severe congenital diseases worldwide are congenital heart diseases (CHD, 0.8%) [140], neural tube defects (NTD, 0.1%) [141] and Down’s syndrome (DS, 0.1%) [142]. At present, the most widely used screening tool for congenital diseases is the anomaly ultrasound scan, performed at around 18–22 weeks of pregnancy [143]. Whilst most NTDs can be reliably detected on an ultrasound, heart defects are more difficult to see and despite improvements in technology the detection rate for CHD is only around 50%, with many not detected until birth [3, 143]. Invasive diagnostic tests, such as amniocentesis or chorionic villi sampling, can be useful to screen for genetic diseases such as DS, but these carry a small but significant risk of miscarriage (0.35%) [144] and so are not routinely offered. Over the last 10 years, efforts have been focused on development of non-invasive prenatal tests (NIPT) based on the analysis of maternal blood which minimise risk to the foetus. A NIPT test for DS based on copy number variant analysis of foetal DNA in the maternal blood is now offered to patients on the NHS [145]. These tests are limited to genetic diseases which can be detected by analysis of DNA; however, only a minority of CHD cases have a known genetic cause [146]. There is a clinical need for a NIPT to detect CHD. Rather than detecting DNA, a more sophisticated real-time test that provides information on the health of the developing foetus is needed. Such a test would detect signals produced by the developing foetus that cross the placenta and can be detected in maternal blood. EVs appear to be good candidates for such a test as they have a complex cargo and can be traced to their cell of origin by surface marker expression.

### Challenges in development of a non-invasive prenatal EV test

#### Biomarker discovery

The first challenge is to identify which part of the EV to focus the search for biomarkers on. Both the surface molecules and cargo are potential sources of biomarkers. Surface markers include general EV biomarkers such as CD63, as well as tissue-specific markers such as PLAP. Many studies have shown that placental-derived EVs are altered in diseases such as pre-eclampsia, gestational diabetes mellitus and intrauterine growth restrictions [86, 89, 90, 92–95], but it is more difficult to convincingly demonstrate changes in foetal-derived EVs in disease. Post-translational modifications such as glycosylation increase diversity of these markers. The cargo also holds potential biomarkers. RNA in particular is highly dynamic, with the potential to reflect subtle and real-time changes in health, and thus has been the focus of several diagnostic biomarker studies. Despite the identification of several EV biomarkers, to date none has been translated into clinical practice.

Biomarker specificity dictates their overall utility as a diagnostic tool. Most studies to date have applied “omic-like” approaches to perform unbiased global screens for changes in biomarkers without any a priori assumptions. An alternative approach might be to focus on specific signalling pathways known to act during foetal development and believed to be downregulated in the adult. A challenge here is that it is known that certain developmentally regulated pathways are reactivated during cancer [147] and this may limit their foetal specificity.

#### Improving the signal:noise ratio

Given that maternal blood is a complex mixture comprised of maternal, placental and foetal EVs as well as other proteins and RNA, a lack of host specificity may limit the detection levels of certain biomarkers. The purification of foetal EVs would reduce the complexity of the sample allowing for an improved signal:noise ratio. This may therefore be a necessary step in any protocol. It may be, for example, that an EV tissue-specific surface biomarker could be used to purify a test sample from maternal blood, followed by an analysis of cargo biomarkers within this simplified test substrate.

#### Assay development and validation

Whereas in rare cases it may be possible to identify a “yes/no” marker (presence/absence of the molecule in the disease state and not in the healthy controls), it seems likely that in most complex diseases changes may involve more subtle changes across a panel of biomarkers. Here, researchers may need to identify a “disease signature”. Such signatures will need to be carefully validated to establish robustness.

#### Translation into clinic

Once a suitable purification and assay protocol has been developed, the last challenge is the translation from the laboratory into the clinic. One challenge will be whether a simple in-house test can be developed for use in the clinic itself, or whether samples will need to be sent off to a specialist facility for processing. An example of a commonly used technique for the quantification of protein biomarkers in bodily fluids in a clinical setting is an immunoassay [148]. Immunoassays are cost-effective, require limited equipment and can be performed by staff in-house, making it the ideal biomarker detection platform especially in the developing world. The existing NHS NIPT for Down’s syndrome involves genomics analysis performed at a central facility, indicating such a procedure would be feasible, but the isolation and analysis of EV samples would likely require a more complex multiple step protocol.

## Conclusion

EVs represent an emerging and previously unappreciated mechanism of maternal-foetal crosstalk during gestation. In this review, we have discussed various clinical*,* in vitro and animal studies which suggest that EVs released from both the foetus and the mother are able to cross the placenta to facilitate this communication. There is a clinical need for a reliable non-invasive maternal blood diagnostic test for congenital disease. The presence of foetal-derived EVs in maternal blood raises the possibility that these could in the future be utilised as diagnostic biomarkers for congenital disease. To date, most studies of maternal blood EVs are limited to a small number of diseases closely linked to placentation problems such as pre-eclampsia and pre-term birth, in which a clear link to trophoblast-derived EVs has been established (reviewed in [11, 149]). However, the evidence presented here suggests that foetal EVs are trafficked across the placenta carrying “disease-specific” cargo and surface epitopes, which could perhaps be harnessed for diagnostic purposes.

## Data Availability

This is not relevant as no datasets were generated or analyzed during the current study.

## Abbreviations

ALIX: ALG-2 interacting protein-X
BDNF: Brain-derived neurotrophic factor
CAG: Chicken beta-actin promoter
CD: Cluster of differentiation
CFDA-SE: Carboxyfluorescein diacetate succinimidyl ester
CHD: Congenital heart disease
CNTN2: Contactin-2
C19MC: Chromosome 19 miRNA cluster
C14MC: Chromosome 14 miRNA cluster
DDAO-SE: Dodecyldimethylamine oxide succinimidyl ester
DiI: 1,1-Dioctadecyl-3,3,3,3-tetramethylindocarbocyanine perchlorate
DiR: 1,1-Dioctadecyl-3,3,3,3-tetramethylindotricarbocyanine iodide
DS: Down’s syndrome
EGFP: Enhanced green fluorescent protein
ER: Endoplasmic reticulum
ESCRT: Endosomal sorting complex required for transport
ESE: Early sorting endosome
EV: Extracellular vesicle
GFP: Green fluorescent protein
GPI: Glycosylphosphatidylinositol
HLA-G: Human leukocyte antigen-G
ILV: Intraluminal vesicle
ISEV: International Society for Extracellular Vesicles
lncRNA: Long non-coding RNA
LSE: Late sorting endosome
LY6E: Lymphocyte antigen 6 family member E
MARCKS: Myristoylated alanine rich C-kinase substrate
mG: Membrane tagged MARCKS-eGFP
miRNA: MicroRNA
mT: Membrane tagged MARCKS-tomato
MV: Microvesicle
MVB: Multivesicular body
NIPT: Non-invasive prenatal tests
NK: Natural killer
NTA: Nanoparticle tracking analysis
NTD: Neural tube defects
PKH26: Paul Karl Horan 26
PLAP: Placental alkaline phosphatase
SNA: Syncytial nuclear aggregate
STBM: Syncytiotrophoblast-derived microvesicle
SOX 2: SRY-box transcription factor 2
TDT: Tandem dimer tomato
TGC: Trophoblast giant cell
Th: Helper T cell
Tc: Cytotoxic T cell
TMC1: Transmembrane channel-like protein 1
TSG101: Tumour susceptibility gene 101
